# Study protocol for a cluster randomized trial of a school, family, and community intervention for preventing drug misuse among older adolescents in the Cherokee Nation

**DOI:** 10.1186/s13063-022-06096-0

**Published:** 2022-02-23

**Authors:** Kelli A. Komro, Terrence K. Kominsky, Juli R. Skinner, Melvin D. Livingston, Bethany J. Livingston, Kristin Avance, Ashley N. Lincoln, Caroline M. Barry, Andrew L. Walker, Dallas W. Pettigrew, Lisa J. Merlo, Hannah L. F. Cooper, Alexander C. Wagenaar

**Affiliations:** 1 Department of Behavioral, Social, and Health Education Sciences, Rollins School of Public Health, Emory University, 1518 Clifton Road NE, Atlanta, GA 30322 USA; 2 Cherokee Nation Behavioral Health, 19600 N. Ross St, Tahlequah, OK 74464 USA; 3 College of Arts and Sciences, Anne and Henry Zarrow School of Social Work, University of Oklahoma, Tulsa, USA; 4 Department of Psychiatry, College of Medicine, University of Florida, Gainesville, USA

**Keywords:** Opioids, Marijuana, Alcohol, Primary prevention, Multi-level intervention, Youth, American Indian, Rural

## Abstract

**Background:**

The national opioid crisis has disproportionately burdened rural White populations and American Indian/Alaska Native (AI/AN) populations. Therefore, Cherokee Nation and Emory University public health scientists have designed an opioid prevention trial to be conducted in rural communities in the Cherokee Nation (northeast Oklahoma) with AI and other (mostly White) adolescents and young adults. Our goal is to implement and evaluate a theory-based, integrated multi-level community intervention designed to prevent the onset and escalation of opioid and other drug misuse. Two distinct intervention approaches—community organizing, as implemented in our established Communities Mobilizing for Change and Action (CMCA) intervention protocol, and universal school-based brief intervention and referral, as implemented in our established Connect intervention protocol—will be integrated with skill-based training for adults to strengthen social support for youth and also with strategic media. Furthermore, we will test systems for sustained implementation within existing organizational structures of the Cherokee Nation and local schools and communities. This study protocol describes the cluster randomized trial, designed to measure implementation and evaluate the effectiveness on primary and secondary outcomes.

**Methods:**

Using a cluster randomized controlled design and constrained randomization, this trial will allocate 20 high schools and surrounding communities to either an intervention or delayed-intervention comparison condition. With a proposed sample of 20 high schools, all enrolled 10th grade students in fall 2021 (ages 15 to 17) will be eligible for participation. During the trial, we will (1) implement interventions through the Cherokee Nation and measure implementation processes and fidelity, (2) measure opioid and other drug use and secondary outcomes every 6 months among a cohort of high school students followed over 3 years through their transition out of high school, (3) test via a cluster randomized trial the effect of the integrated CMCA-Connect intervention, and (4) analyze implementation costs. Primary outcomes include the number of days during the past 30 days of (1) any alcohol use, (2) heavy alcohol use (defined as having at least four, among young women, or five, among young men, standard alcoholic drinks within a couple of hours), (3) any marijuana use, and (4) prescription opioid misuse (defined as “without a doctor’s prescription or differently than how a doctor or medical provider told you to use it”).

**Discussion:**

This trial will expand upon previous research advancing the scientific evidence regarding prevention of opioid and other drug misuse during the critical developmental period of late adolescent transition to young adulthood among a sample of American Indian and other youth living within the Cherokee Nation reservation.

**Trial registration:**

ClinicalTrials.gov NCT04839978. Registered on April 9, 2021. Version 4, January 26, 2022

**Supplementary Information:**

The online version contains supplementary material available at 10.1186/s13063-022-06096-0.

Contributions to the literature Rural Whites and American Indian/Alaska Native (AI/AN) populations are two subgroups that have been hit hard by the opioid crisis, and also experience persistent disparities related to alcohol problems and suicide risk. Early onset of alcohol and other drug use during the adolescent years is a risk for the development of substance use disorder in adulthood.

This study will be the first to test a theory-driven and community-engaged implementation of an integrated school, family, and community drug preventive intervention to target opioid and other drug misuse among older adolescents in the context of rural, American Indian, and multiracial communities within the boundaries of a tribal reservation.

Findings will contribute to gaps in the implementation literature regarding implementation processes and effectiveness of school, family, and community prevention strategies in under-resourced and under-served rural and tribal communities.

## Background

Increased rates of drug- and opioid-involved overdose deaths, along with alcohol-related mortality and suicide, have contributed to an unprecedented rise in midlife mortality—compellingly labeled as “deaths of despair” [1, 2]. Significant and widening disparities exist for subgroups of the population by race/ethnicity, socioeconomic status, and geographic area—disparities driven by adverse economic trends, socioeconomic inequality, and vulnerabilities due to historical trauma, discrimination, and lack of resources [2]. Rural Whites and American Indian/Alaska Native (AI/AN) populations are two subgroups hit hard by the opioid crisis, which have also experienced persistent disparities related to alcohol and suicide risk [2–4].

The COVID-19 pandemic heightened concern for the risk of mental health and substance use problems [5–7]. Indeed, provisional data indicate an alarming 27% increase of drug overdose deaths from September 2019 to September 2020, rising to rates higher than during the previous height of the opioid epidemic in 2017 [8]. Survey data from June 2020 indicate that nearly half of adults reported at least one adverse mental or behavioral health symptom, with the highest levels among young adults aged 18–24 years (75%) and those who held less than a high school diploma (66%) [9]. Young adults reported significant increases in mental health and substance use symptoms during COVID-19 [10].

Primary prevention, by addressing risk and protective factors related to the onset and sequence of mental health symptoms and drug use, is key to reversing the alarming trends and disparities. Early onset of drug use during the adolescent years is a risk factor for faster transition to substance use disorder during adolescence and into adulthood. Adolescents aged 12 to 17 who reported initiation of cannabis or prescription drug misuse during the past year were nearly twice as likely to have a substance use disorder (SUD) than young adults aged 18 to 25 who initiated within the past year [11]. Alcohol and cigarette use preceding initiation of marijuana use remains the most prevalent sequence of drug use onset among youth, but opioid misuse is playing a more prominent role in the development of SUDs among younger generations [12]. Adolescents who engage in simultaneous co-ingestion of opioids and other drugs have greater odds at age 35 of alcohol use disorder, cannabis use disorder, other drug use disorder, and any substance use disorder, relative to those with no history of non-medical opioid use during adolescence [13].

Our trial is focused on universal primary prevention for rural older adolescents living in or near the boundaries of the Cherokee Nation reservation, a 6950 square mile area within 14 counties of northeast Oklahoma. Within the reservation area fixed by treaty in 1866, the Cherokee Nation currently shares jurisdictional authority with the State of Oklahoma and county and municipal governments. Cherokee Nation is the largest federally recognized American Indian tribe with 369,295 registered tribal members. During 2014–2016, within the 14-county region, the age-adjusted rate of overdose death involving opioids ranged from 13 to 36 per 100,000 [14], the lower end of the range being higher than in Oklahoma as a whole (11.6) and similar to the US average (13.3) [15]. Oklahoma has had one of the highest rates of opioid overdose mortality [3], and provisional data indicate that drug overdose deaths rose by 16% from September 2019 to September 2020 [8].

This preventive intervention will be implemented through Cherokee Nation Behavioral Health and builds upon the strengths and services provided by Cherokee Nation Health Services, which is the largest tribally operated health care system in the USA [16]. Cherokee Nation Behavioral Health provides prevention, treatment, and recovery services [17]. Prevention activities include public education, social norm campaigns, training to schools and other community organizations, and individual, family, and group counseling. Treatment services include medically assisted treatment, assistance for inpatient treatment, and wraparound care management for clients and their families. In 2013, Cherokee Nation Behavioral Health started the Helping Everyone Reach Out (HERO) Project. The HERO Project is a comprehensive strategy to improve outcomes for Cherokee (and other American Indian) children and families within the Cherokee Nation reservation by providing a spectrum of community-based services and support for children and youth with or at risk for mental health or other challenges. Cherokee Nation Behavioral Health has emphasized children and youth services and public health approaches to reach an under-served population with high rates of trauma, setting the stage for partnerships with academic scientists to meet challenges with evidence-based community-driven approaches.

In a previous NIH-funded prevention trial in the Cherokee Nation, we found that an alcohol-focused school- and community-based preventive intervention reduced alcohol use among adolescents, but also had beneficial spillover effects that reduced other drug use, including prescription drug misuse [18, 19]. Other prevention trials have also found that involving multiple community systems is effective for the prevention of drug use among adolescents and young adults [20, 21]. Interventions to prevent initiation and progression of alcohol, marijuana, and opioid misuse among rural adolescents need to address barriers to accessing health and social services, facilitators of access to harmful drugs, family and community cohesion, and educational and economic opportunities [2, 22, 23]. Given the multi-level and multi-system determinants of substance misuse, we have designed a multi-level intervention with delivery at the school and community levels.

### Contribution of this trial

This study will be the first to test a theory-driven and community-engaged implementation of an integrated school, family, and community drug preventive intervention to target opioid and other drug misuse among older adolescents in the context of rural, American Indian, and multiracial communities within the boundaries of a tribal reservation. Findings will contribute to gaps in the implementation literature regarding implementation processes and effectiveness of school and community prevention strategies in under-resourced and under-served rural and tribal communities.

### Objectives and aims

The trial will achieve the following aims: (1) implement school-, family-, and community-level interventions through existing structures of Cherokee Nation Behavioral Health and Oklahoma public schools and measure implementation processes and fidelity at the individual, school, and community levels; (2) measure primary drug use and secondary outcomes at the individual level; (3) test the effect of the integrated community (CMCA) and school and family (Connect) intervention; and (4) analyze implementation costs.

## Method

### Trial management

The current trial was funded through the National Institutes of Health (NIH) Helping to End Addiction Long-term (HEAL) Initiative (https://heal.nih.gov/research/new-strategies/preventing-opioid-use-disorder). The NIH HEAL Preventing Opioid Use Disorder in Older Adolescents and Young Adults Initiative (HPI), funded by the National Institute on Drug Abuse (NIDA), supports the development and dissemination of strategies to prevent opioid misuse and opioid use disorder among vulnerable populations of young people. The HEAL Prevention Cooperative (HPC) consists of 10 HPI research projects and a coordinating center that work across a variety of settings and populations to test preventive intervention strategies. The HPI coordinating center (RTI International, principal investigators (PIs) Phillip Graham, Ty Ridenour) facilitates data harmonization, implementation science, cost-effectiveness research, and dissemination of study findings across the 10 HPI projects.

The prevention trial in the Cherokee Nation is led by a team at Emory University and Cherokee Nation Behavioral Health. The following investigators have primary responsibilities in their respective areas of expertise: PI Komro: overall study management, recruitment, intervention refinement, intervention implementation, implementation measurement, outcome measurement, and analysis; PI Skinner: intervention refinement, intervention implementation, and implementation measurement; PI Kominsky: recruitment, outcome measurement, data collection, and analysis; Co-I Wagenaar: community intervention refinement, implementation, implementation measurement, and analysis; Co-I Cooper: community intervention refinement and outcome measurement; Co-I MD Livingston: trial design and analysis; Consultant Merlo: screening and brief intervention and motivational interviewing; Consultant Pettigrew: community intervention refinement and implementation. Project manager BJ Livingston manages all aspects of the trial, including weekly meetings with the PI and intervention team, bimonthly meetings with the evaluation team, and monthly meetings with investigators, including NIDA scientist Dr. Kathy Etz.

### Trial design

**Fig. 1:**
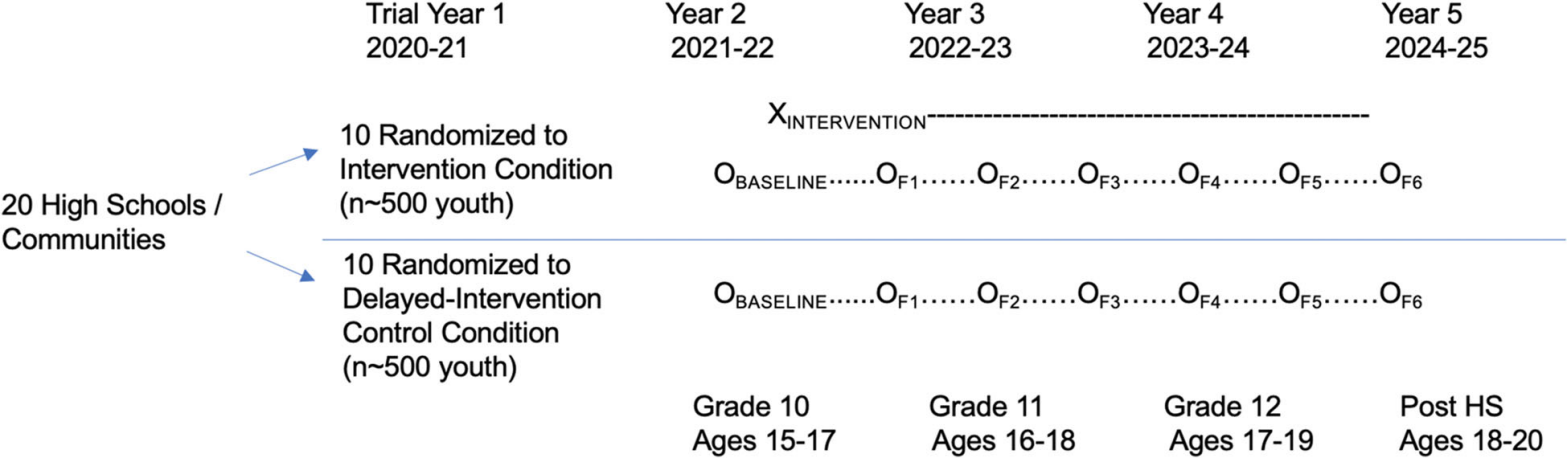
Study design

The study design is a cluster randomized trial (CRT) with one baseline survey and six follow-up surveys (Fig. 1). The intervention condition involves exposure to 3 years of an integrated multi-level intervention within the school and community. The control condition communities will be offered the project intervention materials, resources, and trainings after follow-up evaluation has been completed. Baseline assessment will take place among a cohort of youth during the fall semester of 10th grade (ages 15 to 17) before intervention initiation. Intervention activities will begin during the cohort’s 10th grade year and will continue regardless of school status and for 6 months following on-time graduation. Students who are suspended, expelled, or drop out of high school and remain within the community will continue to be tracked for follow-up surveys. Follow-up surveys will be conducted every 6 months over a 3-year period measuring changes in proximal and distal outcomes as the intervention is initiated, ramps up, and becomes routine. The final follow-up survey will be conducted 6 months after on-time graduation, allowing assessment of changes in drug use outcomes during the important transition from high school to young adulthood.

Human subjects’ approval was received from the Cherokee Nation IRB, which is serving as the single IRB for this multi-site trial involving Cherokee Nation Behavioral Health and Emory University. All data collected will be de-identified and stored on secure servers accessible only to members of the research team. Any adverse events or protocol modifications will be tracked and, when indicated, reported in a timely manner to the institutional review board and funding agency.

### Study setting, participants, and recruitment

**Fig. 2:**
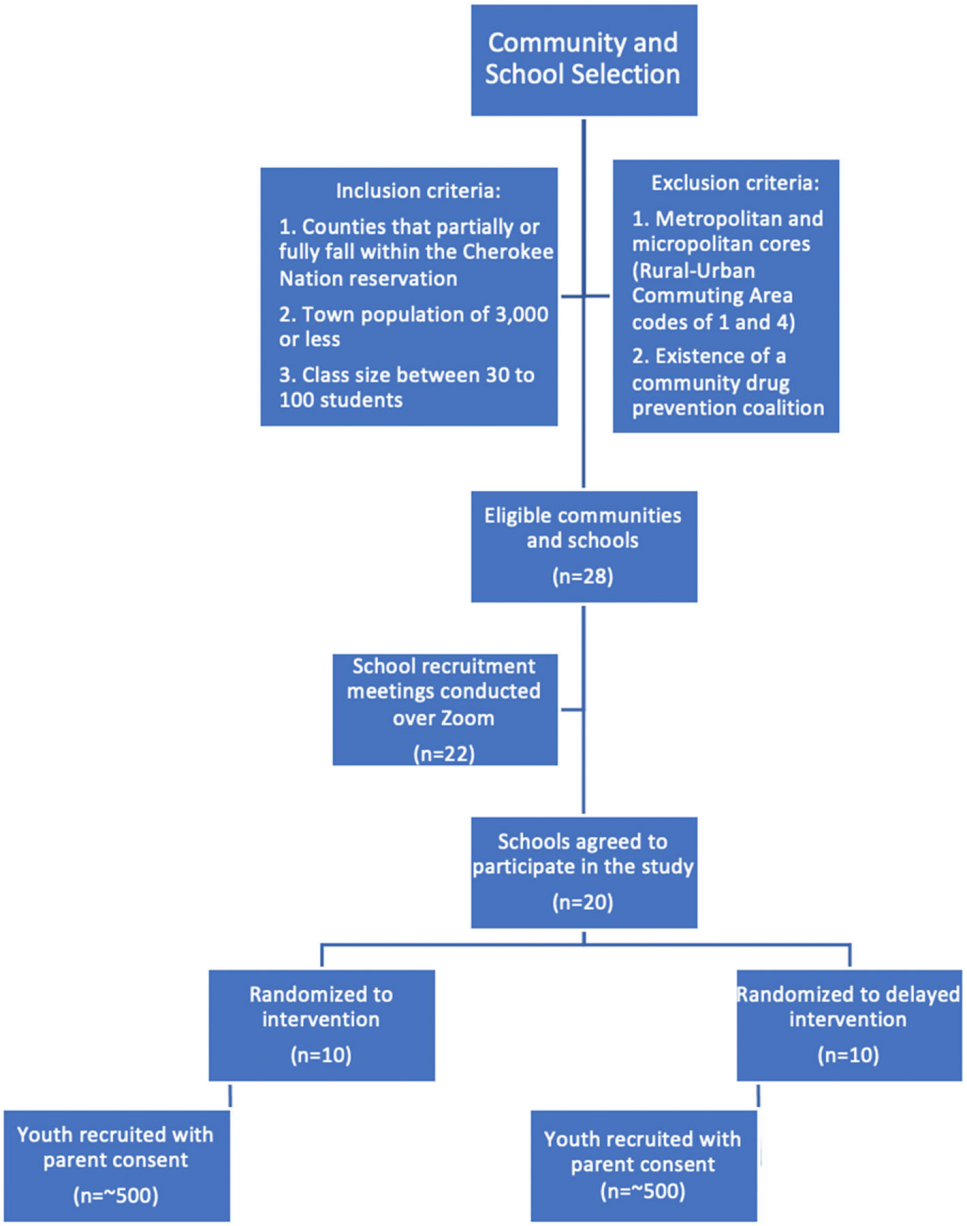
Community and school selection, recruitment, allocation, and participant enrollment

The target population is students attending high schools in small rural towns in the 14 counties that partially or fully fall within the Cherokee Nation reservation. Figure 2 displays a planned flow chart for the study. Inclusion criteria for high schools include (1) counties that partially or fully fall within the Cherokee Nation reservation, (2) town population of 3000 or fewer, and (3) class size between 30 and 100 students. Exclusion criteria include (1) metropolitan and micropolitan cores (Census Bureau Rural-Urban Commuting Area codes 1 and 4) and (2) the existence of an established community drug prevention coalition. Based on the study inclusion and exclusion criteria, 28 of 60 schools met eligibility criteria. Eligible school/community units were selected for the first phase of recruitment based on geographic separation to reduce spillover risks of the community-level intervention. Twenty-four schools were invited to a recruitment meeting; 22 responded and attended a Zoom meeting with the project team. Twenty of the 22 agreed to participate in the study.

All 10th grade students (ages 15 to 17 years) enrolled in the participating high schools during the fall 2021 semester will be eligible to participate in the study. The only exclusion criterion is being unable to understand written or verbal English. Based on historical records from the 20 schools, we will recruit approximately 1000 students. Participating schools will either provide the researchers with a list of the most current home addresses for all students in the grade cohort in the participating high schools or be provided with postage-paid consent letters to mail directly from school. Parent/guardian consent letters describe the study and provide a toll-free telephone number that parents/guardians can call with questions or concerns. Home visits by PI Kominsky or the evaluation coordinator will be made when requested or if the parent is unavailable via telephone. Parents who refuse permission for their child to participate will be asked to respond by returning an addressed postage-paid postcard or by calling Cherokee Nation Behavioral Health or Emory University using toll-free telephone numbers. Also, each student will be given an assent form and opportunity to decide not to participate or withdraw their participation at any time.

A number of steps will be taken to maximize participation and retention rates. The fact that the intervention and assessments proposed in this application will be endorsed by the Cherokee Nation and principals at the participating high schools should help to maximize participation rates. Primary data collection will occur in cooperation with the schools during school time, and youth will receive compensation for their time. We will use procedures for tracking and follow-up surveys including secure Internet and in-person survey administration options. Although some attrition is anticipated, a substantial proportion of the students recruited at baseline are expected to be retained in the study. In our previous prevention trial in the Cherokee Nation, we maintained over 80% participation each year of the 3-year study.

Our recruitment and retention protocol requires persistence, rapport development skills, making multiple contact attempts, tracking all efforts, and speaking directly and courteously with community members and study participants, which has worked well for the investigators’ other school- and community-based studies. We have been able to recruit a sizeable percentage of the sample and experienced few refusals. As with all field studies, recruiting students and parents in this fashion necessarily introduces the potential for selection bias because those who participate may be systematically different from those who decline. However, maintaining a respectable baseline participation rate, conducting descriptive analyses to determine whether the final sample is representative of the school demographically and to ensure comparability on relevant characteristics between communities, and performing analytic operations to respondent data (e.g., weighting to adjust for nonresponse) help offset the impact of selection bias. Also, bias will be somewhat further reduced by providing financial incentives and maintaining direct contact with both parents and students.

### Constrained randomization

Following recruitment of 20 school-based clusters conducted by the PIs (Komro, Kominsky, Skinner) and project manager (BJ Livingston), clusters will be allocated to either the intervention condition or delayed-intervention control condition using constrained randomization by the project statistician (MD Livingston). Constrained randomization helps to ensure balanced cluster sizes as well as similar levels of risk between the intervention and control at baseline [24]. Constrained randomization begins by enumerating all possible randomization schemes (184,756 for 20 clusters with 10 conditions per cluster). Possible randomization schemes are then removed that do not achieve a priori balance conditions, while leaving enough candidate randomization schemes to maintain randomness. Existing guidelines indicate at least 1000 candidate randomization schemes are sufficient for our study design [25, 26]. Finally, one of these randomization schemes is chosen at random. We constrained possible randomization schemes based on balance criteria for both high school and community characteristics (Table 1), resulting in 1226 possible randomization schemes of which one was randomly selected. Following randomization, participating schools will be informed as to their status as intervention or delayed-intervention control schools.

**Table 1:** Variables included in constrained randomization

Characteristic	Balance for constrained randomization	Source
**High school**		
% American Indian	Within 5 percentage points	National Center for Education Statistics [27]
Absenteeism rate	Within 5 percentage points	Oklahoma School Report Cards [28]
% Free/reduced price lunch	Within 5 percentage points	National Center for Education Statistics [27]
Rate of substance use incidents per 100 students	Within 0.5 incidents per 100 students	Oklahoma Department of Education Annual Incident and Suspension Report [29]
Four-year graduation rate	Within 5 percentage points	Oklahoma School Report Cards [28]
Class size	Within 5 students or 10%	National Center for Education Statistics [27]
**Community**		
Medical marijuana dispensary	No more than 1 discrepant community across conditions	OMMA Dispensary List [30]
City police	No more than 1 discrepant community across condition	Google search
Mental health counselors	Mean number per condition within 1	OK State Board of Behavioral Health-LPC License Search [31]

### Preventive interventions

We will implement an integrated community intervention that efficiently combines components from evidence-based strategies at the individual, social network, and community levels, with innovations designed to strengthen and enhance effects. The multi-level intervention is designed to reduce *demand for* and *supply of* drugs. Demand reduction strategies include enhancing social connections and support, building self-efficacy to avoid drugs, and supporting no-use norms. Supply reduction strategies include reducing access to drugs and supporting community norms around protecting youth from access to alcohol and other drugs. The intervention is based on our previous study results, designed as primary prevention, and will be delivered universally and at multiple levels of influence. As such, there will be no special criteria for discontinuing or modifying allocated interventions to individual participants.

#### Connect

The Connect school-based intervention will be administered by the youth services branch of Cherokee Nation Behavioral Health and primarily focus on demand reduction by strengthening self-efficacy, drug-free norms, and social connections and support. One to two Bachelor-level social workers (or closely related degree) will be hired to serve as Connect coaches in the 10 intervention schools. Their direct supervisor will be a Cherokee Nation Behavioral Health licensed clinical social worker who is trained and experienced using motivational interviewing (MI), specifically with American Indian youth. In addition, a PhD-level licensed clinical psychologist (Merlo) who is a member of the Motivational Interviewing Network of Trainers (MINT) will provide initial and ongoing training to the coaches. In each of five school semesters (spring of 10th grade through spring of 12th grade), all students in the study cohort will be invited to complete a computer-based screening and brief intervention for approximately 15 min on a project computer in a private school office or via Zoom. For students habitually absent from school, coaches will schedule sessions via electronic communications (e.g., Zoom, texts, calls, private messages via encrypted social media such as WhatsApp) and conduct face-to-face meetings in private community settings such as Cherokee Nation clinics.

During the Connect session, the computer-based intervention will screen, assess risk, and initiate MI. MI will be used with low-risk students to affirm the student’s decision to avoid drug use and develop personal goals to remain drug-free. For students assessed at moderate or high risk, MI will be conducted to enhance motivation to avoid riskier situations with access to drugs and decrease drug use. Additionally, coaches will have a follow-up appointment within 1 month to monitor each moderate- and high-risk student, adjust goals, normalize setbacks, explore ambivalence about changing relevant behaviors, and provide ongoing support. For high-risk students, a follow-up session with a Connect coach will facilitate connection to substance use and mental health treatment and link students and their guardians to relevant social service resources. The full complement of Cherokee Nation health services is available to any AI youth. Non-Indian youth will be linked with other community services.

Connect coaches will also implement skill training in Youth Mental Health First Aid (https://www.mentalhealthfirstaid.org/about/) for teachers, other school staff, parents, and community members to expand school and community natural helpers and build a network of trusted support for youth. Youth Mental Health First Aid develops skills to identify youth with mental health and substance use concerns, and how to communicate and intervene to provide support.

#### Communities mobilizing for change and action (CMCA)

One to two regional community organizers will be hired to focus on reducing the supply of drugs to youth. Other key goals of CMCA are to empower citizens to make change in their homes and communities, enhance social connections, and support drug-free norms. CMCA organizers will be supervised, guided, and supported by team members with expertise in community organizing and opioid and other drug prevention and harm reduction. Community organizers will attend a series of project trainings, including ongoing skill-based training. Following initial training and baseline data collection, each organizer will initiate the CMCA intervention, which involves educating and organizing of adult volunteers. We will provide trainings and tools, including Family Action Kits, to support local families, community organizations, and citizens. This will include information on national and local opioid and other drug use; evidence-based policies, programs, and practices; and how to motivate and create family and local citizen action for drug prevention. The six-stage organizing process includes (1) assessment of community conditions, norms, and practices; (2) building family and citizen involvement in prevention; (3) enhancing knowledge and building skills for taking preventive actions; (4) developing action steps; (5) implementing prevention actions; and (6) assessing initial results, celebrating accomplishments, and refining planned next steps for maintaining effort and institutionalizing changes. Potential actions will focus on reducing easy access of alcohol and other drugs to youth and may include educational campaigns, behavioral supports, drug take-back initiatives, strategies to reduce social sources of drugs, and collaborations with law enforcement and other community institutions to prevent and reduce easy drug access for youth. The organizing approach is about empowering citizens, including parents, to take preventive actions and to influence relevant institutional leaders to take actions for structural and sustainable change. Strategic planning meetings with project leadership and organizers will occur multiple times a year and incorporate additional training for the community organizers.

#### Media

Media campaigns will support the Connect and CMCA interventions and will include (1) earned media (aka “media advocacy” or generated news coverage); (2) strategically designed, timed, and placed paid media; and (3) social media venues such as Facebook, Twitter, Instagram, and lesser-known locally used platforms. Media campaigns will be designed for different segments of the community, including family and other social networks important to youth.

### Primary and secondary outcomes

#### Data collection

Variables of interest will be measured with a self-report survey of the cohort of youth ages 15 to 17 at baseline and followed over 3 years through their transition out of high school (ages 18 to 20). The first survey will be conducted prior to intervention initiation during the fall of the cohort’s 10th grade year. Six follow-up surveys will occur, one every 6 months, beginning in the spring of their 10th grade year; follow-ups begin immediately following intervention initiation, and end 6 months following high school graduation. Youth surveys will be administered via a secure Web-based survey using project-owned tablets or personal devices. The self-report survey administration will primarily take place in schools, with administration at home or other private locations when youth are not present in school. Survey data collection will be incentivized and will vary by survey location. Youth will be invited to participate in each survey regardless of their participation in intervention protocols.

To provide a high level of reliable data, participants who complete the surveys must have full confidence in the investigators and a high level of trust in the data collectors. One aspect of this trust is the absolute protection of the subjects’ right to confidentiality. All subjects will be assigned a code number when they are enrolled in the study. This number will be used to identify all materials. Furthermore, the data collected in this study will be protected by the use of two databases. The first includes the names and identification numbers. This file is password protected, encrypted, and accessible to only a small number of project staff. The second file includes identification numbers without any names linked with survey responses. This database is available only to those on the project who need access.

For the Web-based surveys and brief intervention, no user responses will be recorded anywhere on the data entry tablet or computer. Study participants will be assigned unique login IDs and passwords, so the system will “know” who it is when they log on. Database access is controlled through domain-level login credentialing. Study participants have access only through the Web application, which permits only data entry for the logged-in individual. Only the PI and project manager will have direct access to the raw data once it has been entered into the database. Data extracted for pre-analysis processing by other software (e.g., SAS) will not contain any personal identifying information.

Quality assurance monitoring of all assessment procedures will be performed on an ongoing basis. Data collectors will be qualified and thoroughly trained in administration of surveys. Oversight of the research activities will be conducted by the PIs, co-investigators, project manager, and evaluation coordinator through one-on-one supervision, weekly meetings, observation, and file review. Daily program operations, staff oversight, training, and quality assurance of protocols for data collection will be monitored by PI Kominsky, the project manager (BJ Livingston), and the evaluation coordinator.

#### Data management

All data collection will take place physically within or near the Cherokee Nation. There will be no data collection at Emory University. Data collected for the study will be transmitted immediately via cellular-connected tablets using Qualtrics to the Emory University server, where checks will be run for data consistency and quality. It is important to note that the schools will not obtain or maintain any individual-level survey results and research staff will not retain any identifying information; thus, there will be no way to link names with survey results. Processes are in place to generate de-identified databases for analyses.

#### Outcome measures

The survey includes common measures developed in collaboration with the HEAL Prevention Cooperative (i.e., 10 research projects and a coordinating center) and were heavily drawn from the PhenX Toolkit on substance use patterns—adolescent module, measured with standard items from the Monitoring the Future (MTF) study [32]. PhenX uses a consensus process and inputs from the scientific community to provide well-established, high-quality, low-burden measurement protocols [https://www.phenx.org/]. Primary outcome measures are number of days during the past 30 days of any (1) alcohol use, (2) heavy alcohol use (defined as having at least four, among young women, or five, among young men, standard alcoholic drinks within a couple of hours), (3) marijuana use, and (4) prescription opioid misuse (defined as “without a doctor’s prescription or differently than how a doctor or medical provider told you to use it”). Change in number of days of substance use will be compared between study arms from baseline to 6 months post-on-time high school graduation.

Secondary outcomes of key mediators (i.e., hypothesized mechanisms) include social support, perceived availability of drugs, social normative beliefs, self-efficacy, and normative estimates. Social support from parents/caregivers, friends, teachers, and community members is assessed with 24 items which are responded to on a 4-point scale where 0 = never and 3 = often. Ease or difficulty in accessing alcohol, marijuana, and prescription opioids is assessed with 12 items using a 4-point scale where 0 = very difficult to get and 3 = very easy to get. Participants will be asked 12 items to assess if they think various social groups disapprove of young people drinking alcohol, using marijuana, and prescription opioid misuse (parents, community adults, peers, self). Responses are given as 0 = do not disapprove, 1 = disapprove, and 2 = strongly disapprove. Self-efficacy is assessed with 4 items asking how easy or hard it would be for participants to ask for help or refuse alcohol or drugs. Responses are given on a 4-point scale, where 0 = very easy and 3 = very hard. Normative estimates of peer drug use (alcohol, marijuana, prescription opioid misuse) are assessed with 3 items asking about how many of their peers in school used drugs in the past year. Possible responses are 0 = none or almost none, 1 = less than half, 2 = about half, 3 = more than half, and 4 = almost all or all. In addition to key outcomes, we will include measures of substance use-related problems (i.e., missed responsibilities, social problems), comorbidities (i.e., depression, anxiety, pain), and possible intervention effect moderators (e.g., age, gender, race/ethnicity, Native American tribal identity, measures of economic and food security).

### Fidelity assessments

Field staff will use laptop computers loaded with research-team-designed management information systems that facilitate their work as well as provide ongoing measures of implementation and progress. Forms include easy-to-use drop-down menus that provide response options for each domain to minimize time burdens associated with completing paperwork and to improve overall efficiency. Students will be given a personal project-generated Connect identification number to access the computer-based intervention; no personal identifying information will be entered into the computer or program. The Connect identification number will allow us to track student participation in the program. In addition, Connect coaches will complete interaction forms following each one-on-one encounter with a student. Coaches will document risk level, behavioral goals, dates of follow-up appointments, and referrals made, if applicable. To assess MI skill among the coaches, during training sessions, coaches will complete mock Connect sessions with an actor who portrays a typical student. As in our previous trial, to measure fidelity, mock encounters will be video recorded, coded, and rated using the Motivational Interviewing Treatment Integrity Coding System [33]. We will survey high school teachers to assess confidence, skills, and practices of strategies covered in the Youth Mental Health First Aid training. CMCA organizers will document details of all community contacts, action team members, meetings, actions/events, and organizing outcomes achieved. Data will be uploaded each week and reviewed by the project team and used in strategic planning meetings with leadership and field staff. Additionally, we will assess other unrelated prevention activities as documented by a key school representative on an annual survey; we do not request participating schools suspend any ongoing prevention activities.

### Cost assessments

In collaboration with the HEAL Prevention Cooperative (HPC) and coordinating center, we will document costs and collaborate on cost-effectiveness analysis. The cost instrument developed with the HPC economic workgroup will be used to capture activity-based costing.

### Measures

Measures evaluate community and school context, implementation outcomes, and youth outcomes including hypothesized mediators and moderators. Table 2 displays all study measures, and Supplementary File 1 provides additional detail for the youth outcomes measures. The survey instruments are available by contacting the corresponding author.

**Table 2:** Study measures by construct

Construct	Measure	Type	Informant	Timing
**Context**				
School	School size, race/ethnic composition, etc.	Record	Record	Year 1
Community	Library, churches, city government, police, etc.	Record	Record	Year 1
**Implementation**				
Connect implementation	Session attended, risk level, behavioral goals, use of MI	Session form	Web-based program, Connect coach	Year 2–4
Connect MI fidelity	Motivational Interviewing Treatment Integrity Coding System	Videotaped coding of Connect coach MI skills	Observer	Year 2–4
Teacher adoption of practices	Confidence, skills, and practices	Survey	Teachers	Year 2–4
CMCA implementation	Contacts, action team members, meetings, actions/events, and outcomes achieved	Access database forms	CMCA organizers	Year 2–4
Other prevention activities	Curriculum, screenings, parent education, community prevention, media, etc.	Survey	School representative	Year 2–4
**Youth measures**				
Demographics	Age, gender, race/ethnicity, tribal citizenship, tribal identity, free/reduced price lunch	Survey	Youth	Year 2–5
Mediators	Social support, perceived availability of drugs, social normative beliefs, self-efficacy, normative estimates	Survey	Youth	Year 2–5
Others	Depression, anxiety, pain, future aspirations	Survey	Youth	Year 2–5
Primary outcomes	Past 30-day use of alcohol, marijuana, prescription opioid misuse	Survey	Youth	Year 2–5

### Data analytic plan

This trial consists of “doubly repeated” measures—repeated measures nested within each student, plus students are nested within schools. To account for both the clustering effect of students nested within schools and repeated measures nested within student, intervention effects on secondary and drug use outcomes will be estimated with generalized linear mixed models specifying a random intercept for each school and allowing within-student residuals to be correlated over time. To estimate change in secondary (e.g., perceived drug availability, norms, social support) and primary (e.g., past month number of days of opioid use without a prescription, marijuana use) outcomes due to the intervention, we will begin by analyzing models of the following form: *g*(*y*) = *β*_0_ + *β*_1_TimeL + *β*_2_IntG + *β*_3_TimeL*IntG where *g*() represents the appropriate link function for outcome *y*, TimeL is a linear variable representing the survey wave, and IntG is an indicator for the intervention group. The parameter of primary interest is *β*_3_, the difference in slope between the intervention and control group. The link function *g*() will be chosen to properly model the distribution for each outcome. If it is found that a linear slope does not fit the shape of the intervention curve well, we will fit models of the following form: *g*(*y*) = *β*_0_ + *β*_1–5_TimeFE + *β*_6_IntG + *β*_7–11_TimeFE*IntG. This model is similar to the previous model except the linear variable for time has been replaced with a series of fixed effects (TimeFE) to allow for a non-linear pattern of change over time. For this model, the parameters *β*_7–11_ must be tested jointly using a likelihood ratio test comparing the proposed model and a model where *β*_7–11_ are fixed to 0. Attrition will be accounted for in all analyses by full information maximum likelihood [34].

Cost analyses will be based on methods described by Miller and Levy [35] and will incorporate the Society for Prevention Research endorsed standards for economic evaluations [36]. Implementation costs will be captured prospectively by including measures of resource use into intervention process evaluation forms. We will collect data on several outcomes: (1) minimum staff to implement the project, (2) local needs (e.g., office space, telephone, local travel), (3) training and technical assistance, (4) opportunity and marginal costs (e.g., support from volunteers), and (5) costs for drug-related problems (e.g., car crashes, emergency department).

### Power

The proposed analytic approach is well-powered to achieve primary study aims. We estimated the statistical power to detect a change in the trajectory over time between intervention and control conditions using PROC GLMPOWER in SAS v9.4. To account for clustering within school and autocorrelation within student, we estimate power for a multivariate model equivalent to our planned “doubly repeated” mixed-effects models previously described. Final multivariate correlation matrices are calculated by taking the Kronecker product of a compound symmetric matrix for students nested within schools (ICC = 0.01 to 0.05) and a LEAR matrix for survey waves nested within student (*ρ* = 0.3, *δ* = 0.2). The specified LEAR structure allows within student autocorrelation to decay more slowly than the AR(1) structure, which is appropriate for adolescent substance use behaviors [37]. To be conservative in our power estimates, we treat time as a fixed effect in all power calculations. Should we determine that linear time trends provide an appropriate fit, power will exceed what is reported below. All power estimates assume a type-1 error rate of 0.05 and 20 balanced schools. While we expect low attrition rates due to planned follow-up procedures, we conservatively assume a 20% attrition rate by specifying the within-school sample size as 46 (an average of 57 10th graders per school times 0.8). Our primary and secondary outcomes are continuous scales or count data. We approximate power for both by treating all outcomes as Gaussian. Assuming a linear intervention effect through post-high school follow-up, we are powered at the 0.8 level to detect a standardized mean difference of 0.32 at an ICC of 0.01 and 0.47 at an ICC of 0.05 at the end of follow-up.

### Monitoring

A Data Monitoring Committee and formal stopping guidelines were not considered as this is a low-risk preventive intervention. The principal investigator will be responsible for monitoring the safety and efficacy of this trial, executing the Data and Safety Monitoring (DSM) plan, and complying with the reporting requirements to the funding agency and Cherokee Nation IRB. The annual DSM report will include the participants’ sociodemographic characteristics, expected versus actual recruitment rates, any regulatory issues that occurred during the past year, summary of adverse events and serious adverse events, and any actions or changes with respect to the protocol. Reporting guidelines will be followed for any unanticipated adverse events and serious adverse events. The investigators have developed a detailed plan for protection of data, mandatory reporting, and addressing a distressed participant that will be applied during the study. There is no anticipated harm for trial participation and no plans for post-trial care. The Cherokee Nation IRB will review and approve all products for dissemination, including presentations and publications.

## Discussion

### Innovation

The innovation of the trial is summarized with three core ideas: combination, integration, and partnership. We combine effective strategies from different levels of influence in socio-ecological models (individual, school, family, community). We combine our longstanding experience and success with adolescent drug prevention with a renewed focus on the developmentally critical (especially for opioid misuse) transition from adolescence to adulthood—from high school to post-secondary education, training, and employment opportunities and into independent adult roles in the community, tribe, and society. We integrate two well-designed, tested, and effective interventions—CMCA and Connect—which to date have been treated as independent interventions (and both found to be effective), into a single mutually reinforcing intervention supported with strategic media. The intervention is theoretically informed, drawing from an integration of diverse theories from psychological, social-psychological, sociological, social work, public policy, and political science fields. This enhances likelihood of improving individuals’ resistance to drug misuse; altering the functioning of social groups; changing structures, procedures, and processes of local institutions; and strengthening community connectedness and anti-drug-misuse norms. Importantly, we administer and oversee implementation of the intervention through Cherokee Nation Behavioral Health, enhancing uptake of the intervention to other sites after the research trial, and substantially improving long-term sustainability. Finally, the proposed trial is innovative because of the depth of partnership. The Cherokee Nation reservation, unlike other reservations, includes citizens of other AI tribes as well as a substantial White population. The intervention will be universally implemented to all, and the project creates the necessary structures and processes to help serve the needs of all (e.g., referrals for special services or treatment). The community environments are complex, with overlapping jurisdictions and responsibilities of the Cherokee Nation with municipalities, counties, school districts, policing agencies, and health systems. The Cherokee Nation has longstanding patterns of partnership across these elements that will be further enhanced during implementation of the trial.

### Limitations

The current study only recruits schools within or near the boundaries of the Cherokee Nation in northeastern Oklahoma, and therefore, the generalizability may be limited to rural communities in or near American Indian reservations. The outcome evaluation is limited to one cohort of youth who will be exposed to the school and community interventions; therefore, the outcomes will not capture potential effects of the community intervention on other youth. The delayed-intervention control group will likely be implementing other prevention activities, which we will measure with an annual survey. Therefore, the effects of the intervention are in comparison to prevention as usual.

## Conclusion and impact

In summary, the proposed trial is significant because it (1) focuses on primary prevention—reducing initiation and early use—rather than secondary prevention to reduce harm among users, (2) focuses on at-risk rural and AI populations, (3) is conducted in close collaboration with the Cherokee Nation (the largest tribe), (4) enhances previously tested and effective CMCA and Connect interventions specifically for opioid misuse and other drug prevention, and (4) evaluates effects and mechanisms of the enhanced intervention using a rigorous randomized cluster trial with well-established measurement and implementation processes.

## Trial status

ClinicalTrials.gov Identifier: NCT04839978. First posted and registered on April 9, 2021; last update posted on January 26, 2022 (version 4). Recruitment will begin in September 2021 and end in August 2024. https://clinicaltrials.gov/ct2/show/NCT04839978

## Supplementary Information

**Additional file 1.** Detailed Measures Table.

**Additional file 2.** SPIRIT 2013 Checklist.

**Additional file 3.** CONSORT 2010 Checklist for Cluster Randomized Trial.

## Data Availability

Please contact the lead author for more information. The researchers at Emory University, in partnership with Cherokee Nation Behavioral Health, agree to securely collect, and store project data and further agree to share the study data, provided all data requests are reviewed and approved by the Cherokee Nation IRB. It is the responsibility of any external party requesting access to project data to request in writing to the project PI the specific data requested and to apply to the Cherokee Nation IRB for such access according to procedures promulgated by the Cherokee Nation. Furthermore, any such access is conditional on meeting all requirements specified by the Cherokee Nation IRB. Trial results will be disseminated through scientific publications, professional conferences, written reports, and in-person and Zoom meetings, and to our community partners and study participants. Cherokee Nation IRB reviews and approves all dissemination products.
