# Supplementary material for: Study protocol for a cluster randomized trial of a school, family, and community intervention for preventing drug misuse among older adolescents in the Cherokee Nation
Source: Trials. 2022 Feb 23;23:175. doi: 10.1186/s13063-022-06096-0 (PMC8864592; doi:10.1186/s13063-022-06096-0)
Supplement: Supplementary file 1 — Additional file 1. Detailed Measures Table. [file 13063_2022_6096_MOESM1_ESM.docx]

**Additional File 1. Detailed youth outcomes measures.**

| **Name/Construct** | **Type (i.e., primary, secondary, other)** | **Description/Instrument** |
| --- | --- | --- |
| Alcohol use (number of days) | Primary | Alcohol use is assessed with a single item that asks the number of days of alcohol use in the past 30 days.[[1]](https://paperpile.com/c/Z2VjY9/WlUj)^,^[^[2]^](https://paperpile.com/c/Z2VjY9/oOVl) |
| Heavy alcohol use (number of days) | Primary | Heavy alcohol use is assessed with a single item that asks the number of days of heavy alcohol use in the past 30 days. Heavy alcohol use is defined as having at least 4 (among young women) or 5 (among young men) alcoholic drinks within a couple of hours.[[1,2]](https://paperpile.com/c/Z2VjY9/WlUj+oOVl) |
| Marijuana use (number of days) | Primary | Marijuana use is assessed with a single item that asks the number of days of marijuana use in the past 30 days.[[1,2]](https://paperpile.com/c/Z2VjY9/WlUj+oOVl) |
| Opioid drug misuse (number of days) | Primary | Opioid drug misuse is assessed with a single item that asks the number of days of prescription opioid misuse in the past 30 days.[[1,2]](https://paperpile.com/c/Z2VjY9/WlUj+oOVl) |
| Social support | Secondary | Social support from teachers,[[3]](https://paperpile.com/c/Z2VjY9/ocFT) parents/caregivers, friends, and community members is assessed with 24 items response options on a 4-point scale where 0 = never and 3 = often. |
| Perceived availability of drugs | Secondary | Ease or difficulty in accessing alcohol, marijuana, and prescription opioids is assessed with 16 items using a 4-point scale where 0 = very difficult to get and 3 = very easy to get.[[1,2,4]](https://paperpile.com/c/Z2VjY9/WlUj+eSxh+oOVl) |
| Social normative beliefs about drug use | Secondary | Participants are asked 12 items to assess if they think various social groups disapprove of young people drinking alcohol, using marijuana, and prescription opioid misuse (parents/caregivers, community adults, peers, and self). Possible responses are indicated on a 3-point scale where 0 = don't disapprove, 1 = disapprove, and 2 = strongly disapprove.[[1,2]](https://paperpile.com/c/Z2VjY9/WlUj+oOVl) |
| Self-efficacy | Secondary | Self-efficacy is assessed with 4 items asking how easy or hard it would be for participants to ask for help or refuse alcohol or drugs in different situations and ask for help when feeling down. Responses given on a 4-point scale, where 0 = very easy and 3 = very hard.[[5]](https://paperpile.com/c/Z2VjY9/GlTw) |
| Normative estimates of peer drug use | Secondary | Normative estimates of peer drug use (alcohol, marijuana, prescription opioid misuse) are assessed with 3 items asking about how many of their peers in school used alcohol, marijuana, and prescription opioids without a doctor’s prescription in the past year. Possible responses are 0 = none or almost none, 1 = less than half, 2 = about half, 3 = more than half, and 4 = almost all or all.[[2,6]](https://paperpile.com/c/Z2VjY9/oOVl+Bool) |
| Depression | Other | Depression is assessed using the PHQ-8 (Patient Health Questionnaire-8).[[7]](https://paperpile.com/c/Z2VjY9/biK2) Items ask how often in the past 2 weeks respondents have been bothered by depressive symptoms such as loss of interest or pleasure, feeling down or hopeless, trouble sleeping or concentrating, and feeling bad about oneself. Possible responses are indicated on a 4-point scale, where 0 = not at all, 1 = several days, 2 = more than half the days, and 3 = nearly every day. Total scores range from 0-24, where scores of 10 or higher indicate major depression, and scores of 20 or higher indicate severe major depression. |
| Anxiety | Other | Anxiety is assessed using the GAD-7.[[8]](https://paperpile.com/c/Z2VjY9/Sce1) Items ask how often in the past 2 weeks respondents have been bothered by anxiety symptoms such as feeling nervous or anxious, uncontrolled worry, trouble relaxing, irritability, and fear of something awful happening. Possible responses are indicated on a 4-point scale, where 0 = not at all, 1 = several days, 2 = more than half the days, and 3 = nearly every day. Total scores range from 0-21, where scores of 10-14 indicate moderate anxiety and scores of 15-21 indicate severe anxiety. |
| Pain | Other | Pain is assessed using 5 items.[[9]](https://paperpile.com/c/Z2VjY9/Kde3) The first item asks respondents to self-report pain intensity in the past 7 days on a scale from 0-10, where 0 = no pain and 10 = worst possible pain. The remaining items ask about frequency of pain interference during the past 7 days. Possible responses are on a 5-point scale, where 0 = never and 4 = almost always. |
| Future aspirations | Other | Future aspirations are assessed with 3 items that ask how important it is that the respondent will 1) graduate from high school, 2) finish college, and 3) have a successful career.[[10]](https://paperpile.com/c/Z2VjY9/z5Wx) Responses are given on a 4-point scale, where 0 = not important and 3 = very important. |
| Age | Demographics | Age is assessed with a single open-ended question that asks, “How old are you?” |
| Gender | Demographics | Gender is assessed with a single item with the following response options: female, male, or decline to answer. |
| Race/ethnicity | Demographics | Race/ethnicity is assessed with two items that ask, “Are you Hispanic or Latinx?” with yes/no/don’t know response options and “What is your race?” with the option to select one or more of the following: American Indian, Alaskan Native, Asian, Black/African American, Native Hawaiian or other Pacific Islander, White/Caucasian, and Other (write-in). |
| Discrimination | Other | Discrimination is assessed with two items that ask about frequency of discrimination on the basis of race/ethnicity and gender. Possible responses are indicated on a 5-point scale, where 0 = never, 1 = hardly ever, 2 = a few times a year, 3 = monthly, and 4 = daily.[[4]](https://paperpile.com/c/Z2VjY9/eSxh) |
| Work/school status | Demographics | Work/school status is assessed with two items that asks respondents to indicate whether they are 1) employed part time, employed full time, not employed and looking for a job, or not employed and not looking for a job, and 2) in school full time, in school part time, or not in school. If in school, a follow-up item asks respondents to indicate their grade/program: 9th grade, 10th grade, 11th grade, 12th grade, working on a GED, enrolled in a technical school or 2-year associate program, enrolled in a 4-year college or university, or other. |
| Free/reduced price lunch | Demographics | If in high school, respondents are asked to indicate whether they receive free or reduced-price lunches at school with the following response options: yes, no, don’t know. |
| Food security | Other | Food security is assessed with 1 item that asks how often in the past 30 days the respondent worried that food at home would run out before their family got money to buy more.[[11]](https://paperpile.com/c/Z2VjY9/jgwE) Possible responses were on a 3-point scale: a lot, sometimes, never. |
| Alcohol/drug use and related problems | Other | In addition to the primary outcome measures, other alcohol and drug use questions include items that assess: 1) lifetime use of alcohol, marijuana, electronic vape products, heroin, methamphetamines, cigarettes, misuse of prescription opioids, and other illegal drugs (yes/no response options and/or number of times used)[[2,12]](https://paperpile.com/c/Z2VjY9/hlR3+oOVl), 2) age of first use[[2,12]](https://paperpile.com/c/Z2VjY9/hlR3+oOVl), 3) use at least a month for 3 months in a row (yes/no), 5) past 12-month missing of work or school, arriving late, getting into trouble, doing a bad job or failing courses because of use of alcohol or drugs (yes/no),[[13]](https://paperpile.com/c/Z2VjY9/5Gos) and 6) past 12-month alcohol or drug-related problems with other people, such as with family members, friends, or people at work such as getting into arguments over use or getting into fights?[[13]](https://paperpile.com/c/Z2VjY9/5Gos) |
| Perceived risk of getting in trouble for use | Secondary | Perceived risk of getting in trouble for using alcohol, marijuana, and prescription opioids without a doctor’s prescription is assessed with 3 items. Possible responses regarding likelihood of getting in trouble for each are on a 4-point scale where 0 = very little chance and 3 = very good chance.[[4]](https://paperpile.com/c/Z2VjY9/eSxh) |
| Tribal citizenship | Demographics | Tribal citizenship is assessed with a single item that asks, “What tribe are you a citizen of?” with the following response options: Cherokee Nation, United Keetoowah Band of Cherokee Indians, Eastern Band of Cherokee Indians, and Other Indian tribe (write-in).[[4]](https://paperpile.com/c/Z2VjY9/eSxh) |
| Tribal identity | Other | Tribal identity is assessed with 3 items from the Multigroup Ethnic Identity scale that ask about one’s sense of belonging, understanding, and attachment towards their tribe.[[14]](https://paperpile.com/c/Z2VjY9/FIt7) Possible responses are indicated on a 5-point scale where 0 = strongly disagree and 4 = strongly agree. |
| COVID-19 impact | Other | The impact of COVID-19 is assessed with a single item that asks, “In the past 12 months, has COVID-19 had an effect on your life?” Possible responses are indicated on a 5-point scale where 0 = extremely negative and 4 = extremely positive.[[2]](https://paperpile.com/c/Z2VjY9/oOVl) |

[1. Johnston, Lloyd D Mietch, R. A. O’Malley, P. M. Bachman, J. G. Schulenberg, J. E. Patrick, M. E. Monitoring the Future national survey results on drug use 1975-2020: Overview, key findings on adolescent drug use [Internet]. Institute for Social Research, University of Michigan; 2021. Available from:](http://paperpile.com/b/Z2VjY9/WlUj) <http://www.monitoringthefuture.org//pubs/monographs/mtf-overview2020.pdf>

[2. Hamilton CM, Strader LC, Pratt JG, Maiese D, Hendershot T, Kwok RK, et al. The PhenX Toolkit: get the most from your measures. Am J Epidemiol. 2011;174:253–60.](http://paperpile.com/b/Z2VjY9/oOVl)

[3. Hanson TL, Kim J-O. Measuring resilience and youth development: the psychometric properties of the Healthy Kids Survey [Internet]. Issues & Answers Report, REL; 2007. Available from:](http://paperpile.com/b/Z2VjY9/ocFT) <https://ies.ed.gov/ncee/edlabs/regions/west/pdf/REL_2007034_body.pdf>

[4. Komro KA, Livingston MD, Kominsky TK, Livingston BJ, Garrett BA, Molina MM, et al. Fifteen-minute comprehensive alcohol risk survey: reliability and validity across American Indian and White adolescents. J Stud Alcohol Drugs. 2015;76:133–42.](http://paperpile.com/b/Z2VjY9/eSxh)

[5. Martin GW, Wilkinson DA, Poulos CX. The Drug Avoidance Self-Efficacy Scale. J Subst Abuse. 1995;7:151–63.](http://paperpile.com/b/Z2VjY9/GlTw)

[6. Arthur MW, Briney JS, David Hawkins J, Abbott RD, Brooke-Weiss BL, Catalano RF. Measuring risk and protection in communities using the Communities That Care Youth Survey [Internet]. Evaluation and Program Planning. 2007. p. 197–211. Available from:](http://paperpile.com/b/Z2VjY9/Bool) <http://dx.doi.org/10.1016/j.evalprogplan.2007.01.009>

[7. Kroenke K, Strine TW, Spitzer RL, Williams JBW, Berry JT, Mokdad AH. The PHQ-8 as a measure of current depression in the general population. J Affect Disord. 2009;114:163–73.](http://paperpile.com/b/Z2VjY9/biK2)

[8. Spitzer RL, Kroenke K, Williams JBW, Löwe B. A brief measure for assessing generalized anxiety disorder: the GAD-7. Arch Intern Med. 2006;166:1092–7.](http://paperpile.com/b/Z2VjY9/Sce1)

[9. Varni JW, Stucky BD, Thissen D, Dewitt EM, Irwin DE, Lai J-S, et al. PROMIS Pediatric Pain Interference Scale: an item response theory analysis of the pediatric pain item bank. J Pain. 2010;11:1109–19.](http://paperpile.com/b/Z2VjY9/Kde3)

[10. Sharp EH, Seaman J, Tucker CJ, Van Gundy KT, Rebellon CJ. Adolescents’ Future Aspirations and Expectations in the Context of a Shifting Rural Economy [Internet]. Journal of Youth and Adolescence. 2020. p. 534–48. Available from:](http://paperpile.com/b/Z2VjY9/z5Wx) <http://dx.doi.org/10.1007/s10964-019-01152-6>

[11. Connell CL, Nord M, Lofton KL, Yadrick K. Food security of older children can be assessed using a standardized survey instrument. J Nutr. 2004;134:2566–72.](http://paperpile.com/b/Z2VjY9/jgwE)

[12. Centers for Diseases Control and Prevention. Youth Risk Behavior Survey Questionnaire [Internet]. 2019 [cited 2021 Jun 1]. Available from:](http://paperpile.com/b/Z2VjY9/hlR3) [www.cdc.gov/yrbs](http://www.cdc.gov/yrbs)

[13. First MB, Williams JBW, Karg RL, Spitzer RL. Structured Clinical Interview for DSM-5 Disorders Clinical Trials Version. Washington, DC: American Psychiatric Association. 2015;](http://paperpile.com/b/Z2VjY9/5Gos)

[14. Roberts RE, Phinney JS, Masse LC, Chen YR, Roberts CR, Romero A. The Structure of Ethnic Identity of Young Adolescents from Diverse Ethnocultural Groups. J Early Adolesc. SAGE Publications Inc; 1999;19:301–22.](http://paperpile.com/b/Z2VjY9/FIt7)
